# Neutrophil side fluorescence: a new indicator for predicting the severity of patients with bronchiectasis

**DOI:** 10.1186/s12890-022-01893-4

**Published:** 2022-03-27

**Authors:** Shiqi Li, Chunxiao Yu, Hongyu Jie, Xinai Han, Shujing Zou, Quanguang Tan, Shugeng Luo, Youming Chen, Jinhong Wang

**Affiliations:** 1 Department of Respiration, The Third Affiliated Hospital, Southern Medical University, Guangzhou, China; 2 Guangdong Provincial Key Laboratory of Bone and Joint Degeneration Diseases, The Third Affiliated Hospital, Southern Medical University, Guangzhou, China; 3 Department of Gastroenterology, The Seventh Affiliated Hospital, Southern Medical University, Foshan, China; 4 Department of Rheumatology and Immunology, The Third Affiliated Hospital, Southern Medical University, Guangzhou, China; 5 Department of Internal Medicine, The Third Affiliated Hospital, Southern Medical University, Guangzhou, China; 6 Department of Clinical Laboratory, The Third Affiliated Hospital, Southern Medical University, Guangzhou, China

**Keywords:** Bronchiectasis, Neutrophil extracellular traps, Neutrophil side fluorescence, Cell population data

## Abstract

**Background:**

Neutrophilic inflammation in the airway is a hallmark of bronchiectasis. Neutrophil extracellular traps (NETs) have been reported to play an important role in the occurrence and development of bronchiectasis. Neutrophil side fluorescence is one of the characteristics of neutrophils that can reflect the activation of neutrophils and the formation of NETs.

**Objective:**

To explore the relationship between the values of neutrophil side fluorescence (NEUT-SFL) in the peripheral blood of bronchiectasis patients, and the severity of the disease.

**Methods:**

82 patients with bronchiectasis from the Department of Respiratory and Critical Medicine, at the Third Affiliated Hospital of Southern Medical University and were scored with Bronchiectasis Severity Index (BSI) (2019–2021). The clinical data such as the value of NEUT-SFL, neutrophil count, C-reactive protein, and procalcitonin levels were collected and retrospectively analyzed. NEUT-SFL values neutrophil count from 28 healthy subjects were also used to ascertain cut-off values.

**Results:**

Based on the BSI scores, patients were divided into three categories as mild (32%), moderate (29%), and severe (39%). Our results showed that the values of NEUT-SFL were higher in bronchiectasis patients compared to healthy controls. The levels of NEUT-SFL positively correlated with the high BSI scores in patients (P = 0.037, r = 0.23) and negatively correlated with the lung function in these patients (r = − 0.35, P = 0.001). The area under the ROC curve was 0.813, the best cut-off was 42.145, indicating that NEUT-SFL values > 42.145 can potentially predict the severity of bronchiectasis.

**Conclusions:**

The values of NEUT-SFL in the peripheral blood can be used for predicting the severity of bronchiectasis.

## Background

Bronchiectasis is a type of chronic bronchial inflammation caused by various factors [1], leading to the destruction of the bronchial wall structure and abnormal and persistent bronchial expansion. Timely antibiotic treatment and regular airway clearance are required for effective treatment of bronchiectasis. Past studies have demonstrated that the inflammatory response can be reduced by controlling chronic bacterial infections, and long-term antibiotic treatment can improve the clinical symptoms [2]. Timely diagnosis and evaluation of the severity of the disease can help in the effective treatment and delay the development of the disease, leading to improved prognosis and quality of life in patients.

Neutrophils have been identified to play a key role in the pathogenesis of chronic pulmonary diseases by releasing the strands of decondensed DNA in complex with histones and a variety of proteolytic enzymes such as neutrophil elastase (NE), collagenase, and metal matrix proteases (MMP-9), called neutrophil extracellular traps (NETs) [3]. The process by which neutrophils produce neutrophil extracellular traps is called NETosis [4]. NETs associated proteins are higher in the sputum of patients with severe exacerbation of bronchiectasis [5]. These proteins have also been found to have a significant correlation with the disease severity, lung function prognosis, and quality of life, indicating that there was an active neutrophilic inflammation inside the airways of patients with severe bronchiectasis [6–8]. The proteolytic enzymes can damage the nearby lung tissues and lead to secondary necrosis of neutrophils, ultimately damaging the airway epithelium and aggravating patients’ clinical symptoms. Studies have also revealed that the levels of NETs associated proteins in the sputum of patients with *Pseudomonas aeruginosa* or *Haemophilus influenzae* infections were significantly higher than those without these infections [5–7]. As the level of NETs increases, the types of microorganisms in sputum decreases [6]. *P. aeruginosa* infection worsens the prognosis of the disease in patients [9–11]. Therefore, NETs promote the occurrence and development of bronchiectasis [12]. At present, there is no specific measure for the assessment of NETs by physicians. Immunofluorescence is used to confirm the existence of NETs, however, it is complicated and time consuming rendering it inconvenient for routine use.

Neutrophil side fluorescence (NEUT-SFL) is one of the techniques used to characterize neutrophils. In this technique, blood cells are analyzed to obtain cell population data (CPD) and various characteristics of blood cells like granularity, volume, and nucleic acid/protein content [13], which reveal the morphology and function of different white blood cells [14]. The Sysmex XN-2000 cell analyzer, used for NEUT-SFL, irradiates cells with a laser wavelength of 633 nm to obtain various CPD [15, 16]. The analyzer applies unique digital calculations and algorithms to classify and count white blood cells, red blood cells, and platelets. In general, the values of SFL mainly reflect the type and quantity of nucleic acids and organelles in the cells. NEUT-SFL is related to the nucleic acid content within the cell [17] and reflects the maturity and activation of neutrophils [18]. NEUT-SFL was found to be significantly correlated with the development of disseminated intravascular coagulation (DIC) in patients with septic shock, indicating that NEUT-SFL could potentially predict sepsis-induced DIC and can be a reliable biomarker indicating activation of neutrophils and NETs formation [19]. However, the exact NEUT-SFL value for predicting the severity of bronchiectasis has not been reported so far. NEUT-SFL values reflect the formation of NETs [20], hence, we speculated that NEUT-SFL might be a potential biomarker for predicting the severity of bronchiectasis in patients and conducted a retrospective study to verify it.

## Methods

### Participants

A retrospective analysis of patients with bronchiectasis who were hospitalized in the Department of Respiratory and Critical Care Medicine in Third Affiliated Hospital of Southern Medical University, China from January 2019 to April 2021 was carried out. A total of 82 cases were included in this study. These patients were admitted to the hospital because of the acute exacerbation of bronchiectasis, such as significantly increased cough and sputum volume. Patients were subjected to HRCT examination and those showing bronchial dilatation in one or more lobes of the lungs were considered to be eligible for enrollment. The exclusion criteria were as follows: 1. Patients who are younger than 18 years; 2. Patients who developed bronchial dilatation secondarily owing to other diseases such as tuberculosis, lung cancer, and pulmonary fibrosis; 3. Patients with incomplete clinical data.

### Data collection

The age, height, weight, lung function, hospitalization history, and the times of exacerbations in the past year, and the scores of mMRC were collected when the patients were admitted to the hospital. As is shown in Table 1, each patient was scored by the Bronchiectasis Severity Index (BSI). BSI scores divided patients into mild (0–4), moderate (5–8), and severe (9–26) [21]. The patients’ peripheral blood parameters including procalcitonin (PCT), C-reactive protein (CRP), and the absolute value of neutrophils (Neu) were collected after admission and before antibiotic treatments. The values of neutrophil side fluorescence of 82 patients and 28 healthy controls were obtained by the Sysmex XN-2000 cell analyzer.

**Table 1:** Characteristics at baseline

Characteristics	All patients (n = 82)	Mild bronchiectasis (n = 26)	Moderate bronchiectasis (n = 24)	Severe bronchiectasis (n = 32)	Healthy control (n = 28)
*BSI score system*					
Sex, n (%)					
Man	41 (50)	7 (27)	14 (58)	20 (62.5)	13 (46)
Woman	41 (50)	19 (73)	10 (42)	12 (37.5)	15 (54)
Age, n (%)					
< 50 y (0)	11 (13)	5 (19)	4 (17)	2 (6)	16 (57)
50 ~ 69 y (2)	50 (61)	20 (77)	13 (54)	17 (53)	11 (39)
70 ~ 79 y (4)	13 (16)	1 (4)	6 (25)	6 (19)	1 (4)
≥ 80 y (6)	8 (10)	0 (0)	1 (4)	7 (22)	0 (0)
BMI (kg/m^2^), n (%)					
< 18.5 (2)	22 (27)	1 (4)	4 (17)	17 (53)	
≥ 18.5 (0)	60 (73)	25 (96)	20 (83)	15 (47)	
FEV1%pred, n (%)					
> 80% (0)	19 (23)	16 (62)	1 (4)	2 (6)	
50% ~ 80% (1)	42 (51)	10 (38)	15 (63)	17 (53)	
30% ~ 49% (2)	15 (18)	0 (0)	6 (25)	9 (28)	
< 30% (3)	6 (8)	0 (0)	2 (8)	4 (13)	
Hospitalization of the last year, n (%)					
Yes (5)	33 (40)	0 (0)	6 (25)	27 (84)	
No (0)	49 (60)	26 (100)	18 (75)	5 (16)	
Times of exacerbations in last years, n (%)					
0 ~ 2 (0)	74 (90)	26 (100)	24 (100)	24 (75)	
≥ 3 (2)	8 (10)	0 (0)	0 (0)	8 (25)	
mMRC score, n (%)					
1 ~ 3 (0)	70 (85)	26 (100)	22 (92)	22 (69)	
4 (2)	12 (15)	0 (0)	2 (8)	10 (31)	
5 (3)	0 (0)	0 (0)	0 (0)	0 (0)	
Pseudomonas aeruginosa infection, n (%)					
Yes (3)	19 (23)	2 (8)	2 (8)	15 (47)	
No (0)	63 (77)	24 (92)	22 (92)	17 (53)	
Other bacterial infections n (%)					
Yes (1)	30 (37)	12 (46)	7 (29)	11 (34)	
No (0)	52 (63)	14 (54)	17 (71)	21 (66)	
HRCT shows the number of dilatated lobes of the lung ≥ 3, n (%)					
Yes (1)	46 (56)	8 (31)	15 (63)	23 (72)	
No (0)	36 (44)	18 (69)	9 (37)	9 (21)	
*Clinical parameters*					
CRP	22.25 ± 42.33	3.40 ± 4.14	25.68 ± 44.97	34.99 ± 51.90	
PCT	0.09 ± 0.17	0.03 ± 0.02	0.09 ± 0.18	0.13 ± 0.23	
Neu^*^	5.20 ± 2.74	4.00 ± 1.41	4.51 ± 1.92	6.70 ± 3.37	3.56 ± 1.00
NEUT-SFL^*^	44.18 ± 3.44	43.47 ± 3.04	44.49 ± 3.23	44.53 ± 3.89	40.42 ± 2.26

Data are presented as mean ± SD except where otherwise indicated. Both sexuality and ages of the two groups had no significance (P = 0.83, P = 0.76, respectively). The level of neutrophil count and NEUT-SFL were statistically different between healthy controls and patients. *P < 0.01

BSI, bronchiectasis severity index; BMI, body mass index; FEV1% pred, the prediction of forced expiratory volume in the first second; mMRC, modified medical research council; CRP, C-reaction protein; PCT, Procalcitonin; HRCT: high resolution computed tomography; Neu, neutrophil; NEUT-SFL, neutrophil side fluorescence

### Statistical analysis

SPSS 25.0 statistical software was used for statistical analysis of the data and Graphpad Prism 8.0.2 for plotting graphs in this paper. The measurement data were expressed by the mean ± standard deviation, and a single-sample SW test was used to test the normality of the data. If the measurement data conform to the normal distribution, the independent sample T-test was performed on the data. Chi-square test and non-parametric test were used for non-normally distributed data and count data. The Pearson correlation analysis method was used for analyzing the correlation between each group of data. Analysis of covariance and Chi square test were used to analyze the effect of age and sex respectively, with P < 0.05 as statistically significant.

## Results

### Analysis of demographic data of patients and healthy controls

Out of the 82 patients, half were men and half female, aged between 25–89 years, with an average age (60.98 ± 14.53) years. A total of 19 patients had *Pseudomonas aeruginosa* infection, while 30 were infected with different bacteria like, *Haemophilus influenzae*, *Staphylococcus aureus*, *Escherichia coli*, *Streptococcus pneumoniae*. and methicillin-resistant *S. aureus*. One of the patients was also found to be coinfected with *H. influenzae* and *S. pneumoniae*. We examined 28 healthy controls, 13 of whom were male and 15 were female, with an average age of 45.11 ± 14.80 years. Statistical analysis showed that the age and sex of patients had no significant effect on the NEUT-SFL. Baseline characteristic are shown in Table 1.

### The level of NEUT-SFL in the peripheral blood of bronchiectasis patients was higher than that of healthy controls

**Fig. 1:**
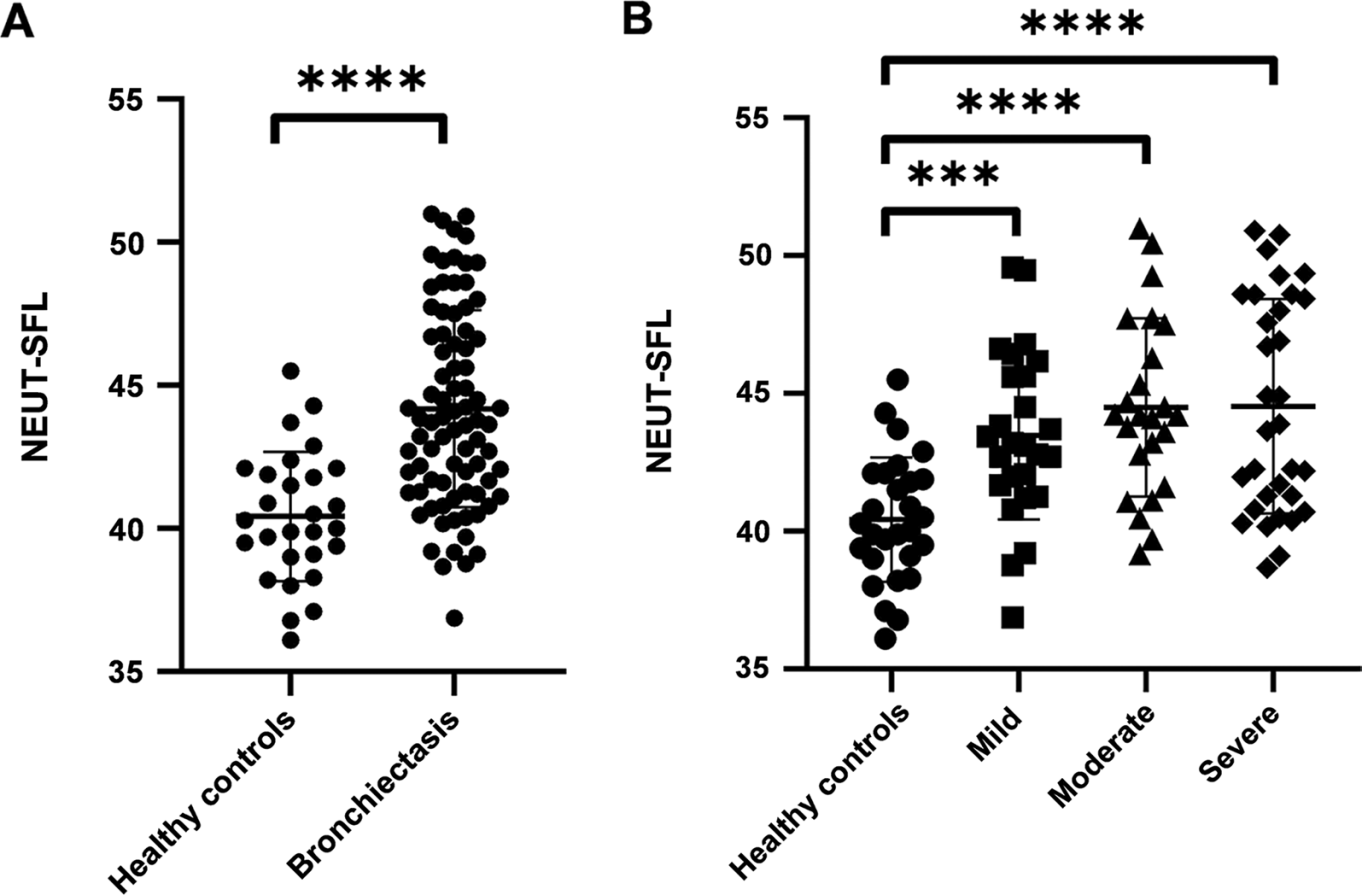
**A** Comparison of the level of NEUT-SFL between patients with bronchiectasis and healthy controls. **B** Comparison of the level of NEUT-SFL between healthy controls and the three groups of patients with bronchiectasis. Healthy controls: n = 28; patients with bronchiectasis: n = 82. ****(P < 0.0001); ***(P < 0.001)

The values of NEUT-SFL in the peripheral blood of bronchiectasis patients were 36.88–50.99 (average value: 44.18 ± 3.44), while the values of NEUT-SFL in healthy controls were 36.10–45.50 (average value: 40.42 ± 2.26). The level of NEUT-SFL in patients with bronchiectasis was higher than that in the healthy controls (Fig. 1A, B).

### The level of NEUT-SFL, CRP and the neutrophil count in the peripheral blood of patients positively correlated with the BSI scores

**Fig. 2:**
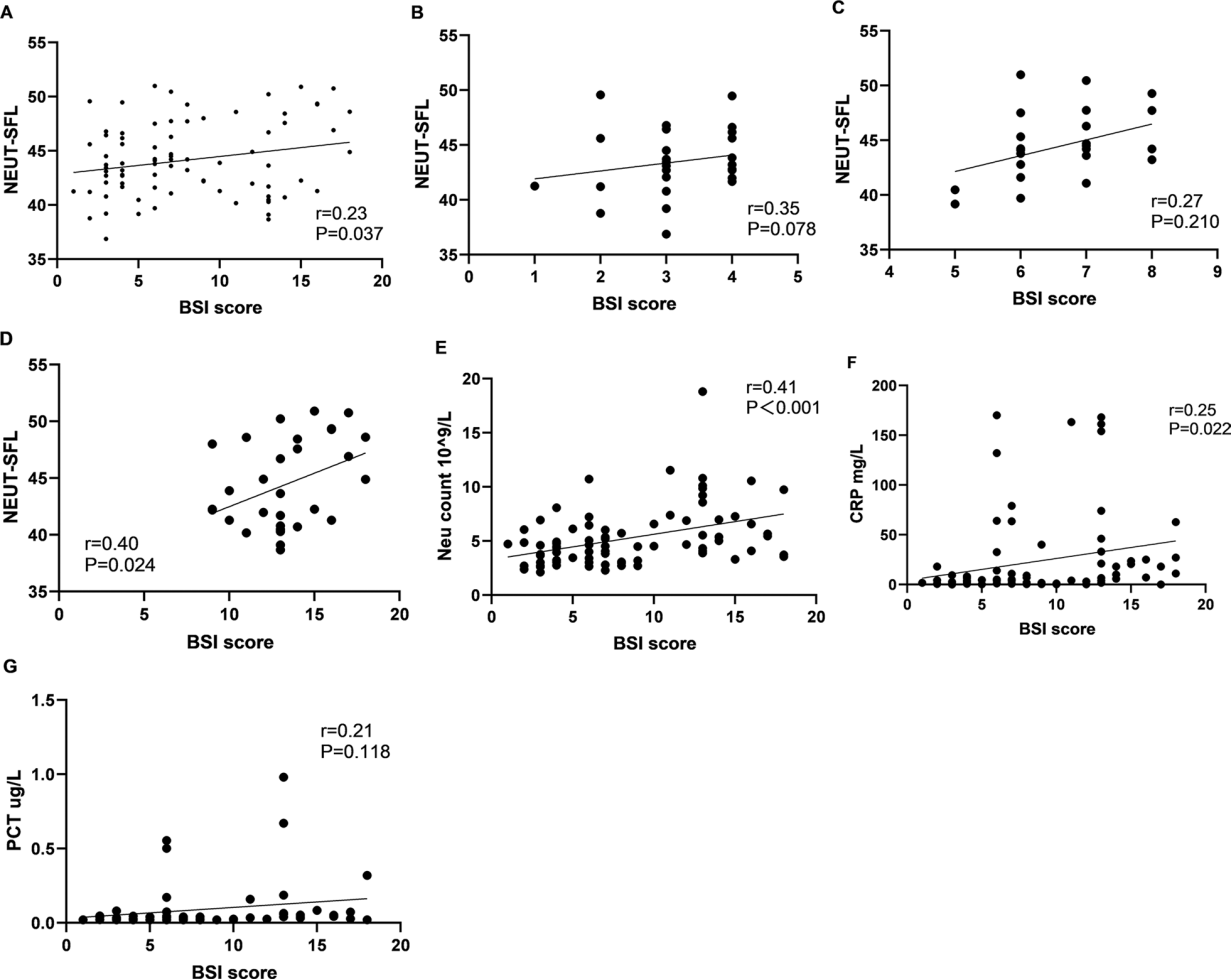
The correlation between BSI scores and the level of NEUT-SFL, neutrophil count, CRP, and PCT. **A** NEUT-SFL in all patients (P = 0.037, r = 0.23); **B** NEUT-SFL in the low BSI group (P = 0.078, r = 0.35); **C** NEUT-SFL in the moderate BSI group (P = 0.210, r = 0.27); **D** NEUT-SFL in the high BSI group (P = 0.024, r = 0.40); **E** The correlation between the level of neutrophil count in the peripheral blood of patients with bronchiectasis and BSI scores (P < 0.001, r = 0.41); **F** The correlation between the level of CRP in the peripheral blood of patients with bronchiectasis and BSI scores (P = 0.022, r = 0.25); **G** The correlation between the level of PCT in the peripheral blood of patients with bronchiectasis and BSI scores (P = 0.118, r = 0.21) (n = 82)

When the entire group (all 82 patients) was considered, the NEUT-SFL values positively correlated to BSI scores (P = 0.037, r = 0.23). We also found that neutrophil count (P < 0.001, r = 0.41) and CRP (P = 0.022, r = 0.25) both positively correlated to the BSI scores, while PCT and BSI scores were not correlated (P = 0.118, r = 0.21). However, when the 3 groups based on BSI score were considered individually, group with high BSI scores showed positive correlation with NEUT-SFL values (P = 0.024, r = 0.40); while no significant correlation was observed in low (P = 0.078, r = 0.35) and moderate (P = 0.210, r = 0.27) BSI score groups (Fig. 2A–G).

### Microorganisms may increase the level of NEUT-SFL in patients with bronchiectasis

**Fig. 3:**
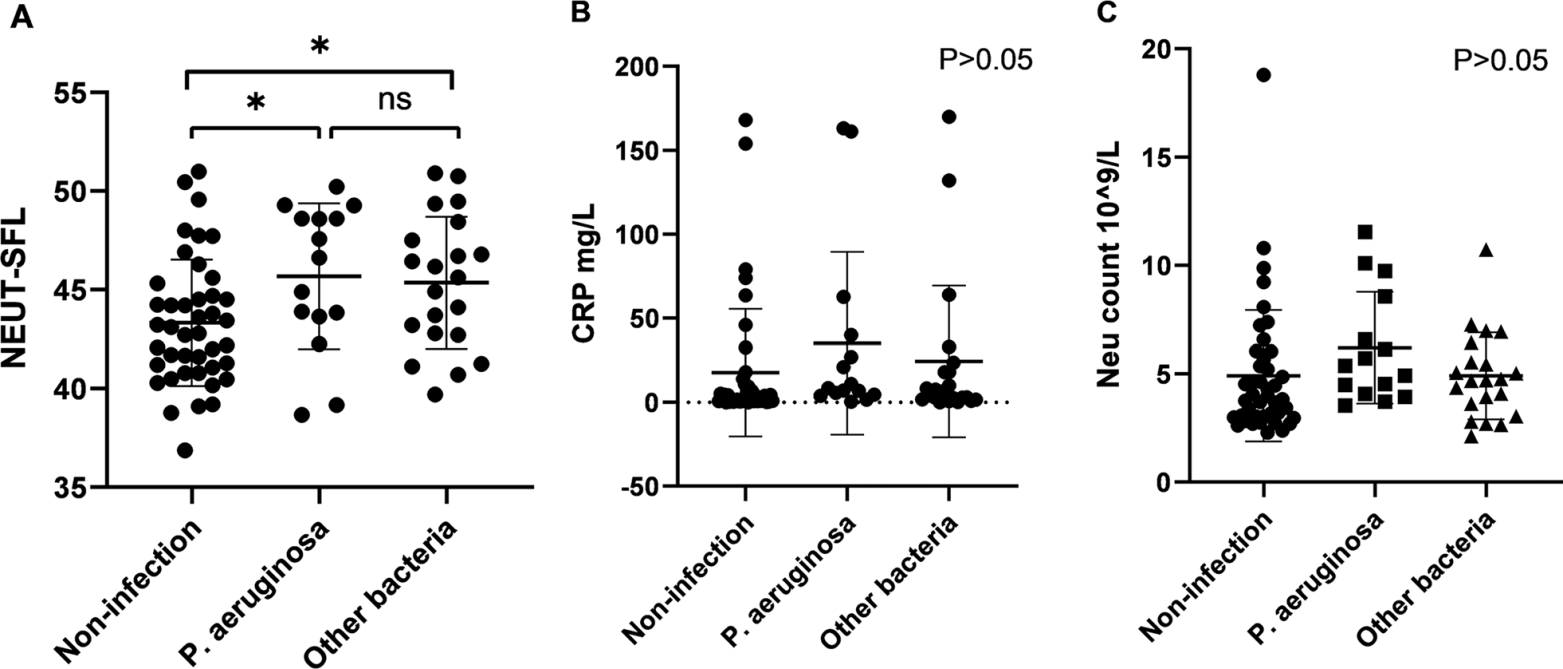
The comparison of the level of NEUT-SFL, CPR and neutrophil count between patients with different bacterial infections. **A** The comparison of the level of NEUT-SFL between patients with *P. aeruginosa*, other bacterial infections and non-infection. **B** The comparison of the level of CRP between patients with *P. aeruginosa*, other bacterial infections and non-infection. **C** The comparison of the level of neutrophil count between patients with *P. aeruginosa*, other bacterial infections and non-infection. (Only P. aeruginosa infection: n = 15; other bacterial infections: n = 21; non-infection: n = 42). *(p < 0.05)

We analyzed the bronchoalveolar lavage fluid from 82 patients with bronchiectasis to estimate the nucleic acid content of respiratory pathogens. The results revealed that only 15 patients presented *P. aeruginosa* infection, and the average value of NEUT-SFL was 45.68. Four patients had > 2 types of bacterial infection, including *P. aeruginosa*, while 42 patients had no bacterial infection. The results indicated that the level of NEUT-SFL in patients with *P. aeruginosa* or other bacterial infection were significantly higher than that in patients with no bacterial infection (P = 0.019, P = 0.023). In addition, there were differences between *P. aeruginosa* infection and other bacterial infections and coinfection respectively (P = 0.021 and P = 0.026, respectively). Neither CRP nor neutrophil count correlated with the microbial species in patients with bronchiectasis (P > 0.05) (Fig. 3A–C).

### NEUT-SFL is negatively related to lung function

**Fig. 4:**
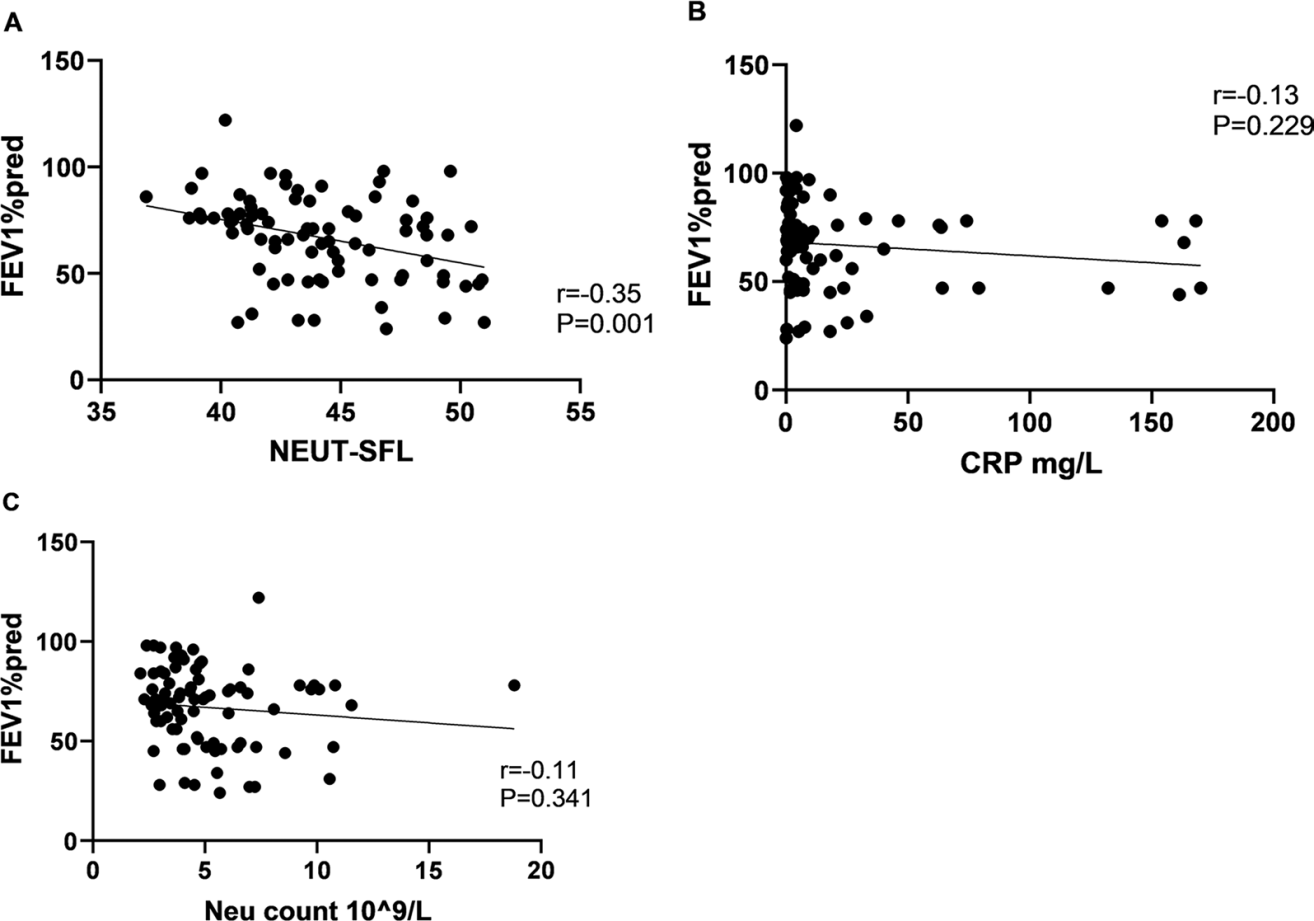
The correlation between FEV1%pred and NEUT-SFL, CRP, and neutrophil count. **A** The level of NEUT-SFL in patients and lung function (P = 0.001); **B** The level of CRP in patients and lung function (P = 0.229); **C** The level of neutrophil count in patients and lung function (P = 0.341; n = 82)

NEUT-SFL in the peripheral blood of the patients with bronchiectasis negatively correlated to their lung function (P = 0.001). However, neither CRP (P = 0.229, r = 0.13) nor neutrophil count (P = 0.341, r = 0.11) correlated with their lung function (Fig. 4A–C).

## Discussion

Bronchiectasis is a chronic airway inflammatory disease. Patients with bronchiectasis have abnormal epithelial remodeling with impaired mucociliary structure, leading to inflammation and infection. In this study, we enrolled patients who were not treated with antibiotics and had no history of oral corticosteroids before admission and hospitalized because of the acute exacerbation of bronchiectasis. We classified patients with bronchiectasis into three groups by using the BSI scoring system and found that the level of NEUT-SFL was elevated in patients, especially in moderate to severe groups. NEUT-SFL, CRP, and neutrophil count positively correlated with BSI scores, indicating that as the neutrophils increased, the infections became more severe. We also found that NEUT-SFL significantly correlated with severe BSI score, indicating that with severe bronchiectasis level of NETs in patients increased, which is consistent with the increase in neutrophil count. However, NEUT-SFL was not correlated with PCT, possibly due to the small samples. To the best of our knowledge, this is the first report revealing that the NEUT-SFL level in patients with bronchiectasis is elevated and is related to the severity of the disease.

**Fig. 5:**
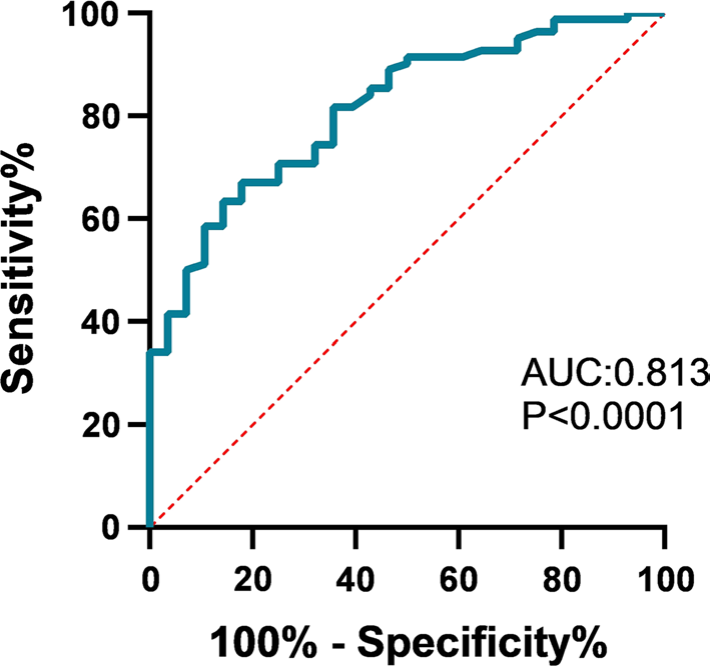
The ROC curve of the level of NEUT-SFL in the peripheral blood of patients with bronchiectasis

Chronic neutrophilic airway inflammation is the main pathophysiological feature of bronchiectasis and plays a key role in the occurrence and development of bronchiectasis. Bronchiectasis exacerbations are usually caused by respiratory infections. Neutrophils are the most abundant immune cell and among the first responders to infection [22]. When there are inflammations in the airways, neutrophils eliminate pathogens by phagocytosis. In addition, neutrophils are activated to undergo NETosis, which release the strands of decondensed DNA in complex with histones and a variety of proteolytic enzymes such as neutrophil elastase (NE), collagenase, and metal matrix proteases (MMP-9), known as neutrophil extracellular traps (NETs) [3], that can capture and kill bacteria and prevent the spread of infection. However, due to a large number of proteolytic enzymes in NETs, the excessive formation of NETs can also damage adjacent lung tissue and the airway matrix, decreasing the efficiency of mucociliary clearance. This in turn leads to bacterial colonization in the airway and ultimately to bronchiectasis [2, 23]. NETs have been reported to play an important role in many chronic respiratory diseases [24] and NE may directly affect the progression of bronchiectasis by promoting mucus production, and emphysema development [8]. For example, the mucus plugs in the airways of patients with COPD increase, and the level of MPO/NETs complex in mucus plugs is negatively correlated with the patients’ lung function, indicating that the extensive formation of NETs leads to airway obstruction [25]. Our results also showed that NEUT-SFL negatively correlated with FEV1%pred, which is consistent with earlier reports. Stiel et al. found that CD66b was the activation marker in neutrophils and it correlated with NEUT-SFL values. NEUT-SFL also reflects the degree of chromatin condensation and serves as a reliable marker of NETosis [19]. Therefore, NEUT-SFL represents the degree of activation of neutrophils. The rapid detection of neutrophils in patients with bronchiectasis can help assess the severity of the condition to provide timely interventions, reduce the clinical symptoms and delay the progression of the disease. CRP, PCT, and neutrophil count are the traditional serological indicators for evaluating infections. Our data showed that PCT was not related to the BSI score, while NEUT-SFL, CRP, and neutrophil count are correlated with the scores. CRP and neutrophil count increase during infections, but also during several other conditions. *P. aeruginosa* is one of the most common bacteria in patients with bronchiectasis. The meta-analysis in the last 5 years has confirmed that *P. aeruginosa* colonization is associated with deterioration of lung function and increased mortality. The clinical outcomes in patients with *P. aeruginosa* colonization are worse [11, 26]. Analysis of the correlations between CRP, neutrophil count, and microbial species related to respiratory disorders in the patients showed that neither of them correlated with the bacterial species. Our results showed that the level of NEUT-SFL was higher in patients with *P. aeruginosa* infection than that in non-infected patients. Although the level of NEUT-SFL of patients with *P. aeruginosa* infection were higher than patients with other bacterial infections, the data showed no statistical significance. We speculate that NEUT-SFL may also be related to the number of bacterial loads in patients, however, further research is required to verify this. In addition, NE activity in the sputum of patients with bronchiectasis is reported to be related to *P. aeruginosa* infection, bacterial load in the airways, and disease severity [8]. However, patients who are diagnosed with dry bronchiectasis have no manifestations of sputum production so that it is difficult to obtain the sputum for the detection of NE activity. This limits the use of NE activity detection in identifying bronchiectasis patients requiring intensive treatments. Compared with the traditional detection of infection parameters such as PCT and CRP in patients, we propose the use of NEUT-SFL to detect the severity of bronchiectasis as this technique takes into account inherent features of neutrophils in the blood routine and can be automatically analyzed by a cell analyzer within 30 min. It is also easier to implement than the sputum NE activity detection method. Under the BSI scoring system, the AUC was 0.813 (95% CI 0.728–0.898) with sensitivity (67.1%) and specificity (82.1%) (Fig. 5). The best cut-off point is 42.145, indicating that NEUT-SFL values above this cut-off may act as a potential biomarker for predicting the severity of bronchiectasis.

We believe that NEUT-SFL is a potential biomarker for clinicians to assess and treat patients with bronchiectasis. First of all, the blood routine test is cheap and it only takes about 20 RMB for this test in China. It can be carried out in every hospital, even in primary hospitals in rural areas. In our opinion, clinicians are able to assess the severity of the disease in patients with bronchiectasis through NEUT-SFL value and give antibiotics to treat the patients according to their own conditions, which is helpful to reduce the times of acute exacerbations. However, this is a preliminary analysis and has some shortcomings. Firstly, all patients enrolled in this study were from the same hospital. It is unclear whether there are statistically significant differences in the peripheral blood NEUT-SFL levels between patients with bronchiectasis and healthy people in different geographical regions. Secondly, there is no correlation between the value of NEUT-SFL of the peripheral blood in patients with bronchiectasis and mild BSI scores. The NEUT-SFL values in the patients with low BSI scores did not increase, possibly due to the limited inflammation in the airways as compared to moderate and high score groups. Thirdly, we only found that NEUT-SFL was elevated in the acute exacerbation of bronchiectasis at present. However, some patients did not return to the hospital for blood tests after discharge, so how quickly do NEUT-SFL changes after infection was well controlled is unclear. As a result, we failed to collected the blood samples from recovered patiens for further analysis at the same time. What’s more. There is no consensus on the duration of prophylactic treatments. Multi-center and large-scale clinical studies are needed to further study different durations of antibiotic prophylaxis.

## Conclusions

In summary, our results suggest that the NEUT-SFL level in the peripheral blood of patients with bronchiectasis is elevated, which may be used as a new peripheral blood biomarker to help determine the severity of disease in these patients and provide timely treatments.

## Data Availability

The datasets used and analysed during the current study are available from the corresponding author on reasonable request.

## Abbreviations

NETs: Neutrophil extracellular traps
NEUT-SFL: Neutrophil side fluorescence
BSI: Bronchiectasis severity index
mMRC: Modified medical research council
HRCT: High resolution computed tomography
NE: Neutrophil elastase
MPO: Myeloperoxidase
CRP: C-reaction protein
PCT: Procalcitonin
*P. aeruginosa*: *Pseudomonas aeruginosa*
ROC: Receiver operating characteristic curve
